# Seasonal simulated photoperiods influence melatonin release and immune markers of pike perch *Sander lucioperca*

**DOI:** 10.1038/s41598-020-59568-1

**Published:** 2020-02-14

**Authors:** Sébastien Baekelandt, Sylvain Milla, Valérie Cornet, Enora Flamion, Yannick Ledoré, Baptiste Redivo, Sascha Antipine, Syaghalirwa N. M. Mandiki, Alexis Houndji, Najlae El Kertaoui, Patrick Kestemont

**Affiliations:** 1 Research Unit in Environmental and Evolutionary Biology (URBE), Institute of Life, Earth & Environment, University of Namur, Rue de Bruxelles 61, Namur, B-5000 Belgium; 2 Animal and Functionality of Animal Products Research Unit (URAFPA), University of Lorraine, Boulevard des Aiguillettes, BP 236, 54506 Vandoeuvre-Les-Nancy, France

**Keywords:** Physiology, Neuroscience, Innate immunity

## Abstract

Melatonin is considered as the time-keeping hormone acting on important physiological functions of teleosts. While the influence of melatonin on reproduction and development is well described, its potential role on immune functions has little been considered. In order to better define an immune modulation by the melatonin hormone, we hypothesized that natural variations of photoperiod and subsequent changes in melatonin release profile may act on immune status of pikeperch. Therefore, we investigated during 70 days the effects of two photoperiod regimes simulating the fall and spring in western Europe, on pikeperch physiological and immune responses. Samples were collected at 04:00 and 15:00 at days 1, 37 and 70. Growth, plasma melatonin levels, innate immune markers and expression of immune-relevant genes in head kidney tissue were assessed. While growth and stress level were not affected by the seasonal simulated photoperiods, nocturnal levels of plasma melatonin were photoperiod-dependent. Innate immune markers, including lysozyme, complement, peroxidase and phagocytic activities, were stimulated by the fall-simulated photoperiod and a significant correlation was made with plasma melatonin. In addition to bring the first evidence of changes in fish immunocompetence related to photoperiod, our results provide an additional indication supporting the immunomodulatory action of melatonin in teleosts.

## Introduction

As photoperiod transducer, the melatonin hormone is mainly produced and secreted by the pineal gland during the night^1–5^. Through this activity, the pineal gland converts light information into a melatonin signal and thus relays information such as the time of the day and year for cells^6,7^. The melatonin hormone is indeed seen as the main actor for anticipating changes in season since its peak of production and release by the pineal organ is directly proportional to the length of the night and thus provides a direct transduction of night length^8^. In mammals as well as in teleosts, melatonin is described to act on important physiological functions, including development and reproduction^3,9–13^. In mammals, it is also known to interact with the immune system^4,12,14–18^. However, such immunomodulatory effects of melatonin have little been investigated in fish. Nevertheless, the available information supports an immune regulation by the melatonin hormone in teleosts^3,19–22^. In pike perch, a potential dual action of cortisol and melatonin hormones on immune defenses was described^23,24^. In addition, these experiments defined a correlation between daily cyclic activities of humoral innate immune markers and the nocturnal peak of plasma melatonin.

The life of the organisms is strongly conditioned by seasons, and this influence is more and more marked further from the equator. Seasonality is known to modulate reproductive activity and to influence food intake, locomotor activity, growth performance and immune responses of teleosts^8,25,26^. In temperate latitudes, the main factors characterizing the seasonal cycle are photoperiod and temperature. From these two factors, the annual cycle of changing photoperiod is the most precise temporal cue for determining the time of year and it has already been well established to influence growth, feeding, smoltification and reproduction^8,25,27,28^. Photoperiod manipulation is also used in aquaculture to modulate sexual maturation and growth of various fish species^29^, including the Eurasian perch (*Perca fluviatilis*) and the pike perch (*Sander lucioperca)*^30^. However, the available information of such influence on fish immune system is very scarce. Considering the potential immunomodulatory action of the melatonin hormone and the annual rhythmicity of melatonin secretion by the pineal gland, it is feasible that changing photoperiod co-ordinates fish immunity through the modulation of melatonin secretion. Since bi-directional communications are described in teleosts between HPI axis and both melatonin axis and immune system, several stress markers were considered in the present experiment. While cortisol is known as a potent immunosuppressive agent in vertebrates^31,32^, a mutual inhibition was described between HPI and melatonin axes^33–35^. In addition, brain serotonergic and dopaminergic activities, which are both indicators of acute and chronic stress in teleosts, were affected by light color and intensity but no consideration of the photoperiod was made^24,35–38^.

Pike perch is the most promising freshwater fish species for the diversification of inland aquaculture industry in Europe. The eyes of this species possess a *tapetum lucidum* that is a specific tissue of the retina which greatly amplify the eye sensitivity to light^24,39^. This is in agreement with behavior of pike perch since it is a crepuscular predator actively feeding during dusk and night^40,41^. Previous experiments have defined a high sensitivity of this species to the light environment. Both light intensity and light spectrum were defined as determining factors affecting its physiology, including endocrine and immune functions^23,40,42,43^. However, as the third light characteristic, photoperiod and its potential effects on fish immunity have still not been considered.

In order to better define in fish the effects of photoperiodic changes on the immunocompetence and the potential key role of the melatonin hormone in this regulation, this study investigated in pike perch the effects of two photoperiod regimes simulating the fall and the spring in western Europe.

## Results

Final body weight (282.8 ± 33.5; 276.5 ± 36.3 g) and specific growth rate (1.04 ± 0.28; 1.12 ± 0.37% d^−1^ for the fall and spring-simulated photoperiod, respectively) did not show any difference between experimental groups. At D70, while the tested photoperiods did not influence the gonadosomatic index for females (0.30 ± 0.04%), a significant difference for males was detected with increased gonadal development with the fall-simulated photoperiod (FSP) (0.41 ± 0.17%) compared to the spring-simulated photoperiod (SSP) (0.22 ± 0.09%). No correlation between immune markers and sexes or gonadosomatic index was tested significant.

**Figure 1:**
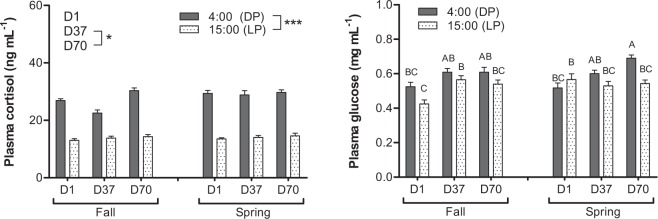
Day-night (DP: dark phase; LP: light phase) variations of plasma cortisol (left) and glucose (right) in pikeperch juveniles exposed during 70 days to photoperiods simulating the fall or the spring in western Europe. Data are expressed as means ± SEM (n = 12). Capital letters indicate significant differences at p < 0.05. Stars (*) and (***) indicate main effects (day of sampling or time of the day) significant at p < 0.05 and 0.001, respectively.

**Figure 2:**
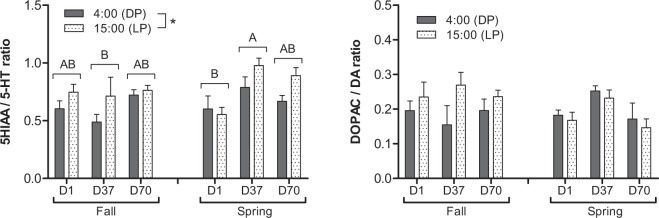
Day-night (DP: dark phase; LP: light phase) variations of brain serotonergic (left) and dopaminergic (right) activities in pikeperch juveniles exposed during 70 days to photoperiods simulating the fall or the spring in western Europe. Data are expressed as means ± SEM (n = 12). Capital letters indicate significant differences at p < 0.05. Stars (*) indicate main effect (time of the day) significant at p < 0.05.

About the tested stress markers, a significant day-night variation was observed for plasma cortisol (p < 0.001), with values reaching 28 ± 4 and 14 ± 2 ng mL^−1^ during the dark and the light phases respectively (Fig. 1). For plasma glucose (Fig. 1), the highest values were observed for the SSP at D70 (4:00) while the lowest were detected for the FSP at D1 (15:00) (p < 0.05). While no effects were observed concerning the dopaminergic activity (Fig. 2), the serotonergic activity was influenced by the time of the day with increases during the light phase (p < 0.05). The SSP also led to an increase in 5HIAA / 5HT ratio from D1 to D37 (p < 0.05) (Fig. 2).

**Figure 3:**
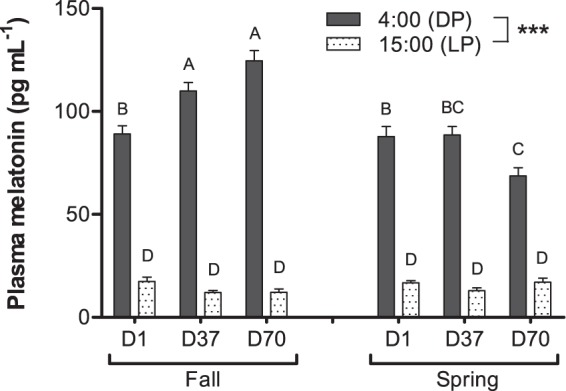
Day-night (DP: dark phase; LP: light phase) variations of plasma melatonin in pikeperch juveniles exposed during 70 days to photoperiods simulating the fall or the spring in western Europe. Data are expressed as means ± SEM (n = 9). Capital letters indicate significant differences at p < 0.05.

While constant during the light phase, plasma melatonin values were significantly influenced by the seasonal simulated photoperiods during the night. It showed a progressive increase for the FSP from 89 ± 14 at D1 to 125 ± 18 pg mL^−1^ at D70 and a decrease for the SSP from 88 ± 17 to 68 ± 14 pg mL^1^ (Fig. 3). No difference was detected between sexes.

**Figure 4:**
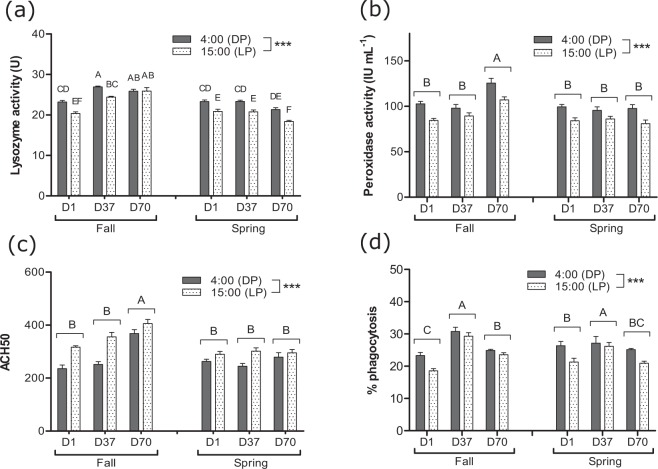
Day-night (DP: dark phase; LP: light phase) variations of (**a**) lysozyme, (**b**) peroxidase and (**c**) hemolytic complement activities in plasma and (**d**) phagocytic activity in spleen of pikeperch juveniles exposed during 70 days to photoperiods simulating the fall or the spring in western Europe. Data are expressed as means ± SEM (n = 15). Capital letters indicate significant differences at p < 0.05. Stars (***) indicate main effect (time of the day) significant at p < 0.001.

Peroxidase and lysozyme activities in plasma and phagocytic activity in spleen all followed a day-night variation with increased activities at 4:00 whatever the simulated photoperiod (p < 0.001) (Fig. 4a,b,d). The opposite scheme was observed for plasma complement activity (Fig. 4c) with the highest values detected at 15:00 (p < 0.001). In addition, lysozyme, peroxidase and complement activities were significantly influenced by the simulated photoperiod with increases observed from D1 to D70 for the FSP. Furthermore, the lysozyme activity significantly decreased from D1 (15:00) to D70 (15:00) for the SSP (p < 0.05). An increase in phagocytic activity was also observed at D37 (p < 0.05) for both photoperiods (Fig. 4d). Significant positive correlations between night variations of plasma melatonin and immune markers were tested significant, including for lysozyme (correlation: 0.41; p < 0.001), peroxidase (0.28; p < 0.01) and complement (0.38; p < 0.01).

**Figure 5:**
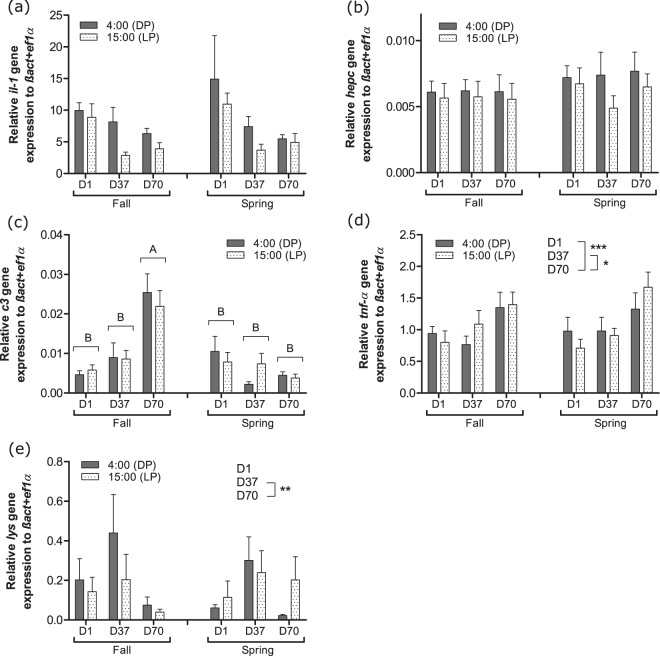
Day-night (DP: dark phase; LP: light phase) variations of (**a**) *il-1*, (**b**) *hepc*, (**c**) *c3*, (**d**) *tnf-α* and (**e**) *lys* gene expression in head kidney of pikeperch juveniles exposed during 70 days to photoperiods simulating the fall or the spring in western Europe. Data are expressed as means ± SEM (n = 12). Capital letters indicate significant differences at p < 0.05. Stars (*), (**) and (***) indicate main effects (day of sampling or time of the day) significant at p < 0.05, 0.01 and 0.001, respectively.

Considering expression of immune-related genes, no effects were detected on *il-1* and *hepc* (Fig. 5a,b). However, *c3* gene expression increased at D70 for the FSP (P < 0.001) (Fig. 5c). In addition, an increase in *tnf-α* gene expression was detected at D70 whatever the experimental group while a decrease in *lys* gene expression was observed at D70 compared to D37 (Fig. 5d,e).

## Discussion

Seasonality, that is mainly characterized by variations of both temperature and day length, is described to affect immune responses of vertebrates, including fish, reptiles, birds and mammals^25,26,44–46^. Thus seasonal variations of several innate immune markers, including complement, peroxidase and lysozyme activities, have been observed in sea bass (*Dicentrarchus labrax*), dab (*Limanda limanda*), halibut (*Hippoglossus hippoglossus*) and Asian catfish (*Clarias batrachus*)^47–50^. Temperature was described several times to be the main seasonal factor operating on fish immune system since lower temperatures lead to a shutdown or slowing of immune mechanisms leading to an increase in fish susceptibility to disease^50–52^. However, the study of Valero *et al*.^50^ has reported that seasonal variations of some immune markers are not related with the temperature and that they could be explained by daylight changes, supporting that both temperature and photoperiod variations act significantly on fish immune system throughout the year. Nevertheless, to our knowledge, no studies conducted specifically on photoperiod in a seasonal context has been published until now. Several studies have focused on photoperiod manipulation (extreme light regimes including constant light or darkness). This practice, that has become common in the aquaculture industry to obtain out-of-season reproductions^25^, affects the levels of stress hormones in rainbow trout (*Oncorhynchus mykiss*), which consequently alter immune functions^29^. It was also shown to affect larval development and survival of European sea bass and Senegalese sole^53,54^.

In the present experiment, the season-simulated photoperiod regimes did not influence growth parameters. Since seasonality was shown to highly influence growth of various fish species^25,49^, our results suggest that such effect is mainly driven by temperature and/or food availability. In addition, values of stress markers suggest that natural gradual changes in L:D are not stressful for pike perch and that it should still be considered in management strategies of fish culture. Since tryptophan is a precursor of both melatonin and serotonin hormones^35^, the daily rhythm in 5HIAA/5-HT ratio may be the result of a decrease in tryptophan availability during the dark phase of the photoperiod due to melatonin synthesis.

An early gonadogenesis was observed at day 70 for both sexes, with the highest development for males maintained under the FSP. The decrease in day length is involved in the initiation of gametogenesis in many fish species including the walleye (*Sander vitreus*)^55^ and the Eurasian perch^56^. Our results are also consistent with results from Ben Ammar *et al*.^57^ showing that the initiation of gonadogenesis seems to be mainly driven by photoperiodic changes. However, both temperature and photoperiod variations must be considered in the control of pikeperch reproductive cycle^57,58^.

A potential effect of such gonadal development on the immune system cannot be discarded since interactions between neuroendocrine and immune systems are widely accepted^59,60^. Moreover, season-dependent changes in fish immune system were shown to be correlated with changes in the levels of circulating sex hormones^61^. For example, phagocytosis and lysozyme activities, respiratory burst activity in blood leukocytes as well as ROS and NOS production are all influenced by estrogens in various fish species. However, in the present experiment, the correlation study does not highlight any relation between immune markers and gonadal development, supporting that immune status of young pike perch may be only slightly influenced by the initiation of gonadogenesis.

We showed for the first time the variations of immune markers according to the season-simulated photoperiod. Lysozyme, complement and peroxidase activities, as well as *c3* gene expression in head kidney, all increased following the decrease in day length during 70 days. In contrast, only lysozyme showed a decrease under the spring-simulated photoperiod. In addition, the correlation study support that variations of innate immune markers are related to the nocturnal variations in plasma melatonin. The progressive exposure to shorter photoperiods leads to an increase in melatonin production and release by the pineal gland since its activity is directly proportional to the length of the night^8^. Few evidences have suggested that the melatonin hormone may act as an important immune regulator in teleosts^3,20^. For instance, several immune markers including peroxidase, phagocytic ability and cytotoxic activity of head kidney leucocytes as well as the expression of immune genes in head kidney (*il-1β*, major histocompatibility complex, and interferon*-*regulatory factor*-*1) are increased in gilthead seabream (*Sparus aurata*) following intraperitoneal injection of melatonin^20^. And, in zebrafish, endogenous melatonin enhances neutrophil migration following increase in cytokine expressions such as Il-8 and Il-1β^22^.

Our results defined also day-night variations of lysozyme, peroxidase, complement and phagocytic activities. Such cyclic activity has already been described in several fish species including pike perch, Nile tilapia (*Oreochromis niloticus*), gilthead seabream and sea bass^19,23,24,62^. The day-night variations of immune variables, as observed in the present experiment, are comparable to the variations of both plasma melatonin and cortisol^23,24^. Considering that cortisol is a potent immunosuppressive agent with complex actions on immune cells and tissues^31,32^ and that an anti-stress role for melatonin has been defined^33,35^, the present results may support that melatonin acts on immune activities through the modulation of cortisol release in plasma^3,63^. However, cortisol peak was described to occur at different times of the day according to the experiment and the fish species, supposing that it is, at least partially, light- and melatonin-independent. Innate immune markers are also differently stimulated according to the time of the day. Even if the underlying mechanisms are still not well understood and that differences of cyclic pattern are observed among fish species^19,23,24^, there are always one or several innate immune activities enhanced whatever the time of the day, ensuring a constant immune protection against pathogens.

All in all, the daily rhythms of innate immune markers and the up- and down-regulations according to the photoperiod support a significant effect of the melatonin hormone on the innate immune system of fish. However, the model of action still needs investigations. Melatonin could act on immune cells and tissues indirectly through the regulation of several hormones (glucocorticoids, growth hormone, prolactin…) or through specific melatonin receptors^3^. In several teleost species, three high-affinity receptor subtypes were identified, including MT1, MT2 and Mel1c^64^. While several studies focused on melatonin receptor distribution^65–68^, few has examined their presence in immune cells or tissues. They are found in the kidney and the spleen of several fish species^66,68,69^ but no study has investigated the exact location of these receptors in the immune tissues neither their functional significance.

In addition, the present study did not examine the potential role of an internal clock machinery in immune modulation according to seasonal simulated photoperiods. Organisms, including fish, show circadian rhythms (repeating roughly every 24 h) of activity, food intake, body change color, oxygen consumption and some physiological parameters^3,70^. These rhythms are under the control of different environmental cues, with the light being the strongest of these synchronizers. Other environmental parameters changing daily or annually, including temperature and food availability, are also critical^70^. The main characteristic of these circadian rhythms is the persistence of their oscillations for a certain period of time, even in the absence of these environmental cues, by being driven by a circadian clock^3^. In zebrafish, a complex network of coexisting central and peripheral clocks was described and peripheral tissue pacemakers, that have been identified in several extra-retinal/extra-pineal tissues, were shown to be directly responsive to light^71^. However, while several studies described daily variations of immune activities^19,23^, none has investigated in fish, and to our knowledge, circadian activity of the immune system or peripheral clock in immune tissues. The potential presence of such pacemaker in immune tissue should be investigated in order to better describe the relationship between the photoperiod, the clock machinery and the immune system.

In conclusion, this study showed for the first time in a teleost fish an innate immune modulation according to the seasonal and daily variations of photoperiod. As the time-keeping hormone, melatonin is seen as one of the main mediator acting on fish immune system. Such regulation may involve both direct and indirect action of melatonin on immune targets. Better consideration of the light environment is suggested to improve immunocompetence of cultured fish species and to limit disease outbreaks.

## Material and Methods

### Animals and rearing conditions

The experiment was carried out at the Aquaculture Experimental Platform (AEP, registration number for animal experimentation C54-547-18) belonging to the URAFPA lab and located at the Faculty of Sciences of the University of Lorraine (France). All experimental manipulations were carried out in agreement with the European and French national legislations on animal welfare after evaluation and approval of the experimental project (protocol number: APAFIS10285-201706201445413) by the local ethic committee in France (Name: CELMEA; French code: 066). A stock of 1,500 mixed-sex pike perch juveniles was provided by Asialor farm (Dieuze, France) and transferred to the facilities. Animals were randomly distributed into 12 indoor 2000-L tanks. Each of these 12 experimental units was operating independently in a recirculating system (RAS). Fish were acclimated for 29 days in constant conditions (temperature: 21 °C; light intensity: 15 lx; photoperiod: LD 12:12) and fed once daily at 2% biomass. They reached 149 ± 21 g at the first day of the experiment. In order to simulate the fall and the spring light conditions in Western Europe, gradual changes in LD from 12_(8:00–20:00)_:12 to 8(10:21–18:16):16 or 12(8:00–20:00):12 to 16(7:25–23:30):8, respectively, were set up for 70 days according to natural photoperiods in Paris, France. Additionally, a dusk and a dawn of 30 min were programmed. Every other rearing condition was maintained constant during all the 70-day experiment. In order to limit high size heterogeneity due to a high social dominance observed in pike perch, fish were fed once a day during the light phase, 2.5 h after sunrise.

### Sampling procedures

Samplings at days 1, 37 and 70 occurred during scotophase at 04:00 h and photophase at 15:00 h. To avoid stress artefacts of nocturnal fishing on diurnal samplings, the number of tanks was doubled. Each of the 4 treatment groups, considering the two simulated photoperiods and the two sampling times, had thus 3 replicates. Five fish were removed randomly from each tank and anesthetized with MS-222 (150 mg L^−1^). Blood was quickly collected by caudal vein puncture with heparinized syringes within 4 min and centrifuged at 3,000 g during 10 min at 4 °C. Fish were then euthanized before collecting the spleen, the whole brain and the anterior kidney. Plasma, brain and anterior kidney were directly frozen in liquid nitrogen and stored at −80 °C until assayed. Spleen Spleen was stored on ice in L-15 media.

Final individual weight and specific growth rate were determined on day 70 for each experimental condition. Specific growth rate was estimated according to the formula: ((Ln (final individual weight) – Ln (initial individual weight)) *100/duration of the experiment). Mortality was recorded along the whole experiment.

Since a slight gonadal development was observed for both males and females at D70, the gonadosomatic index (%) was estimated according to the formula: (gonad weight * 100/body weight).

### Stress indicators

#### Plasma cortisol and glucose

Cortisol was assayed in triplicate using a cortisol ELISA kit (DRG, EIA-1887), following the manufacturer’s instructions (BioSource, Belgium). The intra-assay coefficient of variation was 3.6%, the assay dynamic range was between 0–800 ng mL^−1^ and the analytical sensitivity was 2.5 ng mL^−1 ^^23^. Plasma glucose, also assayed in triplicate, was determined calorimetrically based on a glucose oxidase/peroxidase method described by Trinder^23,72^.

#### Brain neurotransmitters

High Performance Liquid Chromatography (HPLC) was performed according to the methods of Lepage *et al*.^73^, with some modifications^24^, to assess in whole brain the serotonergic and dopaminergic activities expressed as hydroxyl-indol-acetic acid (5-HIAA)/serotonin (5-HT) and 3,4-dihydroxyphenylacetic acid (DOPAC)/dopamine (DA) ratios, respectively.

### Melatonin content in plasma

As described in Baekelandt *et al*.^24^, plasma melatonin was assayed in triplicate using a Melatonin ELISA kit (E-EL-M0788, Elabscience Biotechnology Co., USA), following the manufacturer’s instructions. Recovery rate was estimated around 90 to 95% for melatonin values ranging from 5 to 100 pg mL^−1^. Intra and inter-assays of coefficients were 5.8 and 7.4%, respectively (n = 4). Nocturnal plasma samples were diluted to get values between 5 and 100 pg mL^−1^.

### Humoral immune variables

The total peroxidase activity in plasma was assessed following the method described in Quade and Roth^74^. One unit of peroxidase activity corresponds to an absorbance change of 1 OD.

The alternative complement pathway (ACH50), as described in Baekelandt *et al*.^23^, was assayed by measuring the haemolytic activity in plasma samples using rabbit erythrocytes as targets^75^. Briefly, 10 µL of rabbit red blood cells suspension suspended at 3% in veronal buffer (Biomerieux, Marcy-l’Etoile, France) were mixed with serial dilutions of plasma (from 40 to 800 times). Plates were then read at 615 nm after incubation at 28 °C for 120 min. The spontaneous hemolysis was obtained by adding veronal buffer to 10 µL of rabbit erythrocytes and total lysis was obtained by mixing 10 µL of rabbit erythrocytes to distilled water (total volume = 70 µL). ACH50 corresponds to the lysis of 50% of the rabbit erythrocytes.

Lysozyme activity was evaluated in plasma samples by the turbimetric method^76,77^. Lysozyme activity (units) is defined as the amount of enzyme decreasing the turbidity of 0.001 OD per min.

### Phagocytic activity

Spleen tissues were washed once with L-15 medium. They were then gently mashed with 9 mL of L15 medium supplemented with bovine serum albumin (10%, Sigma-Aldrich) and Penicillin– streptomycin (P/S) (1%, Sigma-Aldrich) through a 100 µm nylon mesh grid. Cell suspensions were kept at 4 °C for 16 h and then centrifuged and washed twice with L15 medium. Cells were suspended in 1 mL of L-15 medium containing 1% of P/S.

Cell mortality was assessed by flow cytometry with a FACSVerse (BD Biosciences, USA) using propidium iodide (PI) probe (1 μg mL^−1^). Samples were considered for analysis when survival exceeded 90%. For the phagocytosis assay, 1 × 10^6^ cells were incubated with yellow-green fluorescent latex beads (Fluoresbrite®, Polyscience; 2 µm diameter) for 18 h at 21 °C with a 1/100 ratio cell-beads ratio. Non-ingested beads were eliminated following a centrifugation step (400 × g, 10 min, 4 °C). Cells were fixed with a 0.5% formaldehyde and 0.2% sodium azide PBS fixating solution. The phagocytic activity (percentage of cells that have ingested three or more beads) was measured through cytometric analysis^78^.

### Gene expression analysis

Total RNA isolation from anterior kidney tissue, which is important in hematopoiesis and immunity in fish, was performed using Extract*-*all® Reagent (Eurobio) following manufacturer’s instructions and description in Baekelandt *et al*.^24^. Each RNA sample was subjected to DNase treatment (DNase Ambion; Life Technologies) and reverse*-*transcription (RevertAid™ H Minus First Strand cDNA Synthesis Kit; Thermo Scientific) following the manufacturer’s instructions. The relative expression of several immune*-*related genes was investigated by RT*-*qPCR, including genes involved in bactericidal defense, namely C*-*type lysozyme (*lys*), hepcidin c (*hepc*), and complement C3 (*c3*), and in pro*-*inflammatory action, namely interleukin*-*1 (*il-1*) and tumor necrosis factor alpha (*tnf-α*). In addition, expression of reference genes *β-actin* and elongation factor alpha (*ef1-α*), whose expressions were tested stable in experimental conditions, were assessed. Efficiencies of primers (Table 1)^24^ were validated when ranged between 90 and 105%. The relative mRNA levels of *c3*, *lys*, *il-1*, *hepc*, and *tnf-α* in each sample were normalized with the geometric mean of *ef1-α* and *β-actin* calculated by the relative standard curve method^79^.

**Table 1:** Sequences of primers used for gene expression quantification^24^.

Gene	GenBank accession #	Sens	Sequence (5′ to 3′)
*β-actin*	MF472627	Forward	CGACATCCGTAAGGACCTGT
		Reverse	GCTGGAAGGTGGACAGAGAG
*ef1- α*	MF472628	Forward	TGATGACACCAACAGCCACT
		Reverse	AAGATTGACCGTCGTTCTGG
*tnf-α*	MK167462	Forward	CTGATTCGCCTCAACGTGTA
		Reverse	GGAGATGGGTCATGAGGAGA
*hepc*	MK036790	Forward	CCGTCGTGCTCACCTTTATT
		Reverse	GCCACGTTTGTGTCTGTTGT
*il-1*	MK036791	Forward	TTTCCCATCATCCACTGACA
		Reverse	ATTCACACACGCACACCATT
*c3*	MF472630	Forward	TGGTGATGTGAGAGGAGCAG
		Reverse	GACGTCATGGCAACAGCATA
*lys*	MF472629	Forward	AGCCAGTGGGAGTCGAGTTA
		Reverse	CATTGTCGGTCAGGAGCTCA

### Statistical analysis

Data are expressed as the mean ± standard error (SEM). Kolmogorov and Smirnov’s test was used to assess the normality of data sets (p < 0.05) and Bartlett’s test was conducted to evaluate variance homogeneity (p < 0.05). Logarithmic transformations were made to achieve normality and homoscedasticity when necessary. Results were analyzed with a three-way ANOVA (p < 0.05) taking the photoperiod regime (fall and spring), the day of sampling (D1, D37 and D70) and the time of the day (4:00 and 15:00) as factors. Statistics were performed using the fish as the experimental unit with the exception of growth parameters (final individual weight and specific growth rate). Tank effect was previously tested not significant. When interactions were tested significant, values were compared according to Tukey’s HSD post-hoc test (p < 0.05). In addition, correlations between immune markers and gonadosomatic index as well as between immune activities and night variations of plasma melatonin were tested for significance. The results were analyzed with JMP 12.1 software (SAS Institute Inc., North Carolina, USA) and graphs were performed with GraphPad Prism V5.04 (California, USA).

## Data Availability

The datasets generated during and/or analysed during the current study are available from the corresponding author on reasonable request.
